# Requirement of Transmembrane Domain for CD154 Association to Lipid Rafts and Subsequent Biological Events

**DOI:** 10.1371/journal.pone.0043070

**Published:** 2012-08-14

**Authors:** Nadir Benslimane, Ghada S. Hassan, Daniel Yacoub, Walid Mourad

**Affiliations:** Laboratoire d’Immunologie Cellulaire et Moléculaire, Centre Hospitalier de l’Université de Montréal, Hôpital Saint-Luc, Montréal, Quebec, Canada; Institute of Molecular and Cell Biology, Singapore

## Abstract

Interaction of CD40 with CD154 leads to recruitment of both molecules into lipid rafts, resulting in bi-directional cell activation. The precise mechanism by which CD154 is translocated into lipid rafts and its impact on CD154 signaling remain largely unknown. Our aim is to identify the domain of CD154 facilitating its association to lipid rafts and the impact of such association on signaling events and cytokine production. Thus, we generated Jurkat cell lines expressing truncated CD154 lacking the cytoplasmic domain or chimeric CD154 in which the transmembrane domain was replaced by that of transferrin receptor I, known to be excluded from lipid rafts. Our results show that cell stimulation with soluble CD40 leads to the association of CD154 wild-type and CD154-truncated, but not CD154-chimera, with lipid rafts. This is correlated with failure of CD154-chimera to activate Akt and p38 MAP kinases, known effectors of CD154 signaling. We also found that CD154-chimera lost the ability to promote IL-2 production upon T cell stimulation with anti-CD3/CD28 and soluble CD40. These results demonstrate the implication of the transmembrane domain of CD154 in lipid raft association, and that this association is necessary for CD154-mediated Akt and p38 activation with consequent enhancement of IL-2 production.

## Introduction

CD154, also known as CD40 ligand (CD40L), is a 33-kDa type II transmembrane protein that belongs to the tumour necrosis factor (TNF) superfamily [1]. CD154 is mainly expressed on activated T cells and platelets and also on a wide range of cell types including dendritic cells, mast cells, monocytes, macrophages, fibroblasts, endothelial cells and others [2], [3]. In addition to interacting with its classical receptor CD40, CD154 was recently demonstrated as a ligand for the integrins α_IIb_β_3_ [4], α_5_β_1_ [5] and Mac-1 [6], [7].

While its interaction with CD40 expressed on antigen-presenting cells (APCs) is clearly important for humoral immune response [8], previous studies indicate that a reciprocal stimulation of T cells might also occur. Indeed, CD154 costimulation was shown to enhance IL-2-mediated T cell proliferation and generation of cytotoxic cells [9]. Other biological functions of CD154 stimulation include potentiation of IL-4 production in CD3-CD28 activated T cells [10]. Also, costimulating T cells via CD3 and CD154 was shown to enhance synthesis of IL-10, TNFα and IFNγ, and at later stages to promote apoptosis [11]. Various signaling events were shown to be initiated when CD154 is stimulated. Activating CD154 molecules on T cells induces phosphorylation of phospho lipase Cγ, and subsequent Ca^2+^-dependent activation of PKC [12]. In addition, CD154 stimulation was shown to induce phosphorylation of MAPKs such as JNK and p38, downstream of Src kinases and Rac-1 activation [13]. It was demonstrated that CD154 signaling mainly JNK, p21-activated kinase 2 as well as NFκB activation was enhanced by an association of CD154 with a splice variant of CD28 in human T cells [14].

Despite the above-described intracellular signaling events triggered via CD154, mechanisms underlying CD154 signaling are still unclear given that the cytoplasmic tail of CD154 is devoid of any signaling motif. We have recently demonstrated that engaged CD154 translocates to lipid rafts to subsequently activate PKCα and PKCγ, and induces the phosphorylation of p38 MAPK. The importance of lipid rafts in the initiation of these CD154-mediated responses was demonstrated using cholesterol-chelating agents to biochemically disrupt lipid rafts [15]. The role of lipid rafts in CD154-mediated activation of PKC and p38 MAPK is not surprising given the importance of lipid rafts in the CD154 system as a whole. Lipid rafts are plasma membrane domains acting as platforms for many signaling effectors, mediating as such signal transduction processes involving insulin receptors, integrins, FcεRI receptor, B cell and T cell receptors, and others [16]. We have previously outlined the importance of lipid rafts for the dimerization of ligated CD40, a process required for the initiation of some CD40-mediated signaling events [17]. Also, we have recently shown that translocation to lipid rafts is necessary for CD40 to induce CD80 expression on B cells via an Akt-dependent mechanism [18].

Our present study aims at identifying the CD154 domain required for its association to lipid rafts and providing direct proof for the involvement of lipid rafts in CD154 signaling using molecular techniques such as mutagenesis. Data presented herein demonstrate that the transmembrane domain of CD154 is responsible for its translocation to lipid rafts and that CD154 signaling is not required for such translocation. Moreover, we further outline the role of lipid rafts in CD154 intracellular signaling and reveal that CD154-induced IL-2 production also necessitates a CD154/lipid raft association.

## Results

### CD154 Cytoplasmic Domain and Intracellular Signaling are not Required for the Association with Lipid Rafts

**Figure 1:**
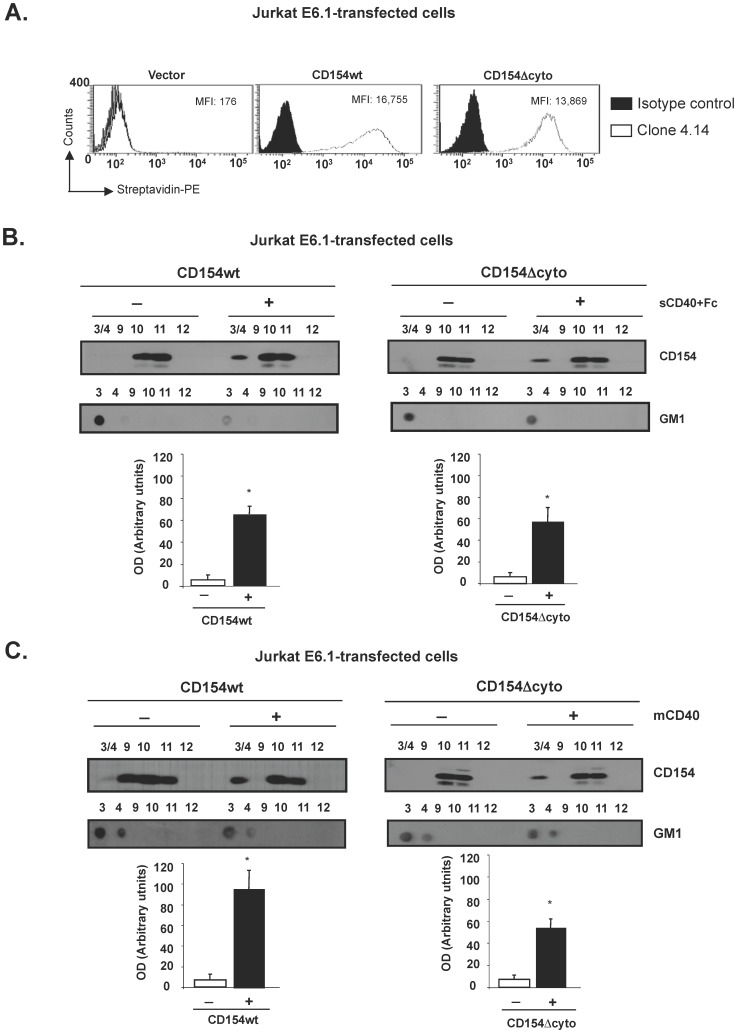
The translocation of CD154 into lipid rafts is independent of its cytoplasmic domain. Jurkat E6.1 cells were stably transfected with vector alone (vector), CD154wt (CD154wt) or with CD154 truncated (CD154Δcyto). *(A)*. Surface expression of CD154 was determined by flow cytometry using the biotin-conjugated anti-CD154 mAb (white plot) and as an isotype control, the biotin-conjugated anti-CD20 mAb IF5 (black plot). Mean fluorescence intensity (MFI) values are indicated. *(B)*. Jurkat E6.1 stably transfected with CD154wt (CD154wt) or with CD154 truncated (CD154Δcyto) were incubated with or without sCD40-Fc (250 ng/10^6^ cells) for 10 min at 37°C. Cells were lysed and subjected to sucrose density gradient. Fractions corresponding to the lipid rafts (3 and 4) were pooled and analysed by immunoblotting as well as fractions corresponding to the soluble domain (9–11). *(C)*. E6.1-CD154wt or E6.1-CD154Δcyto, were co-cultured with murine A20 B cells expressing vector (unstimulated) or human CD40 (membrane-bound CD40, mCD40) for 10 min at 37°C. Then cells were lysed and fractioned as described in A. For the lipid raft localisation, fractions were dot blotted for GM1 utilizing cholera toxin subunit B-HRP. Histograms from B) and C) represent the mean ± SEM of four independent experiments expressed as arbitrary units of optical density (OD) from fractions 3/4 of both unstimulated (−) and sCD40-stimulated (+) cells.

In order to investigate whether CD154 translocation into lipid rafts requires an intracellular signaling, and to determine if the cytoplasmic domain of CD154 is involved in such translocation, we used Jurkat E6.1 cells (a human T cell line negative for CD154) transfected with an empty vector, CD154 wild type (CD154wt), or a truncated form of CD154 lacking its cytoplasmic domain (CD154Δcyto). Fig. 1A shows the expression of CD154 in sorted transfected cells. These cells were unstimulated or stimulated with sCD40-Fc, and then assayed for CD154/lipid raft association, using the sucrose density gradient centrifugation. The low density fractions 3 and 4 represent the triton-insoluble membrane components including lipid rafts, while the detergent-soluble proteins are present in the high-density fractions 9 to 11 [19]. The localization of lipid rafts in fractions 3 and 4 was confirmed by a dot blot analysis identifying the lipid raft marker, glycosphingolipid GM1 in these fractions (Fig. 1B). All CD154 from unstimulated cells were localized into fractions 10 and 11. Upon ligation with sCD40-Fc, a portion of CD154wt is observed in the pooled fractions 3 and 4, suggesting a translocation of the ligated CD154wt into lipid rafts (approximately 13-fold increase in OD in comparison to unstimulated cells). Similarly, CD154Δcyto localized in part in fractions 3 and 4 upon its engagement with sCD40-Fc (approximately 10-fold increase in OD in comparison to unstimulated cells). Similar results were also obtained with cells lyzed with 1% NP-40 (data not shown), indicating that both reducing agents (Triton X-100 and NP-40) produce comparable data.

Stimulation of CD154 was also undertaken using membrane-bound CD40 (mCD40). All CD154 in unstimulated Jurkat E6.1 cells was localized in fractions 9, 10 and 11. As with the soluble stimulus, upon ligation with mCD40, a portion of CD154wt as well as of CD154Δcyto was observed in the pooled fractions 3 and 4, suggesting a translocation of the ligated CD154wt and CD154Δcyto into lipid rafts (approximately 17-fold increase in OD for CD154wt and 10-fold increase for CD154Δcyto). Also, the localization of the lipid raft marker GM1 in fractions 3/4 was used as a positive control (Fig. 1C). Taken together, these results confirm our previously reported data demonstrating a translocation of CD154 into lipid rafts upon its stimulation [15], indicate that such translocation does not involve its cytoplasmic domain and does not therefore require CD154 intracellular signaling.

### The Transmembrane Domain of CD154 is Required for its Association to Lipid Rafts

**Figure 2:**
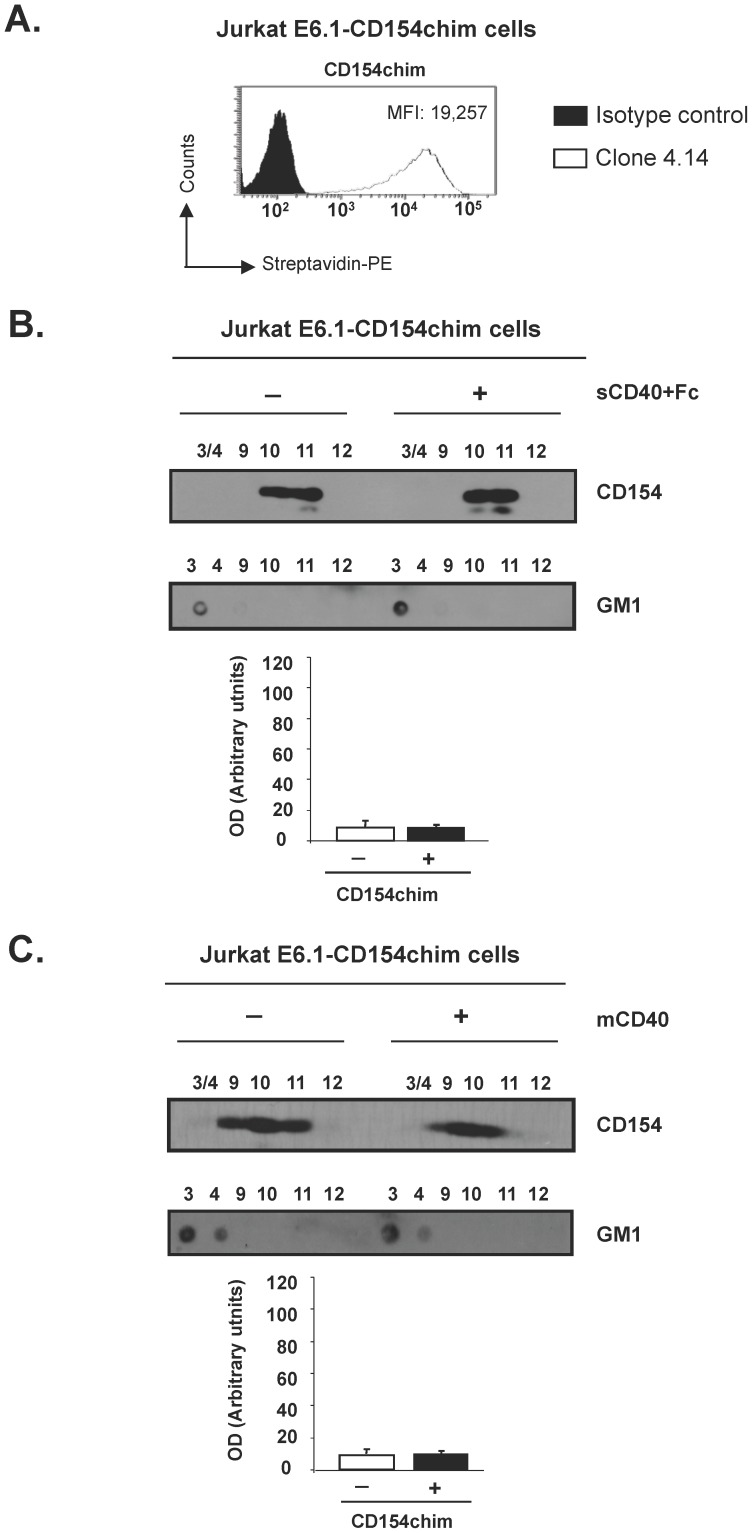
The transmembrane domain of CD154 is mediating the translocation of the molecule into lipid rafts. Jurkat E6.1 stably transfected with CD154 chimera (CD154chim) were used. *(A)*. Surface expression of CD154 was determined by flow cytometry using the biotin-conjugated anti-CD154 mAb (white plot) and as an isotype control, the biotin-conjugated anti-CD20 mAb IF5 (black plot). Mean fluorescence intensity (MFI) value is indicated. *(B)*. Jurkat E6.1 stably expressing CD154chim were incubated with or without sCD40-Fc (250 ng/10^6^ cells) for 10 min at 37°C. Cells were lysed and subjected to sucrose density gradient. Fractions corresponding to the lipid rafts (3 and 4) were pooled and analysed by immunoblotting as well as fractions corresponding to the soluble domain (9–11). *(C)*. E6.1-CD154chim cells, were co-cultured with murine A20 B cells expressing vector (unstimulated) or human CD40 (membrane-bound CD40, mCD40) for 10 min at 37°C. Then cells were lysed and fractioned as described in A. For the lipid raft localisation, fractions were dot blotted for GM1 utilizing cholera toxin subunit B-HRP. Histograms from B) and C) represent the mean ± SEM of four independent experiments expressed as arbitrary units of optical density (OD) from fractions 3/4 of both unstimulated (−) and sCD40-stimulated (+) cells.

Since CD154 is a transmembrane protein, and having demonstrated that the cytoplasmic domain is not involved in its association to lipid rafts, we presumed that the transmembrane domain of CD154 might be involved in such association. To confirm this hypothesis, we generated Jurkat E6.1 cells stably transfected with a chimeric form of CD154 containing the transmembrane domain of transferrin receptor I, another type II protein that is usually excluded from lipid rafts (CD154chim). Figs. 1A and 2A show the expression of CD154 in sorted transfected cells. These cells were unstimulated or stimulated with sCD40-Fc, and then assayed for CD154 translocation into lipid rafts,. The localization of lipid rafts in fractions 3 and 4 was confirmed by a dot blot analysis showing glycosphingolipid GM1 in these fractions (Fig. 2B). As described above, all CD154 from unstimulated cells were localized into fractions 10 and 11. Interestingly, CD154chim was not capable of translocating into lipid rafts upon ligation with sCD40-Fc and remained localized in fractions 10 and 11, as shown in Fig. 2B. Similar results were obtained upon stimulation with membrane-bound CD40 (mCD40). All CD154 in unstimulated Jurkat E6.1 was localized in fractions 9, 10 and 11. The CD154chim was not capable of translocating into lipid rafts upon ligation with mCD40 and remained localized in fractions 9, 10 and 11 (Fig. 2C). In addition, the localization of the lipid raft marker GM1 in fractions 3/4 was used as a positive control (Fig. 2C). Taken together, these results identify the CD154 transmembrane domain as the region mediating CD154/lipid raft association.

### Translocation of CD154 into Lipid Rafts is Required for Intracellular Signaling

**Figure 3:**
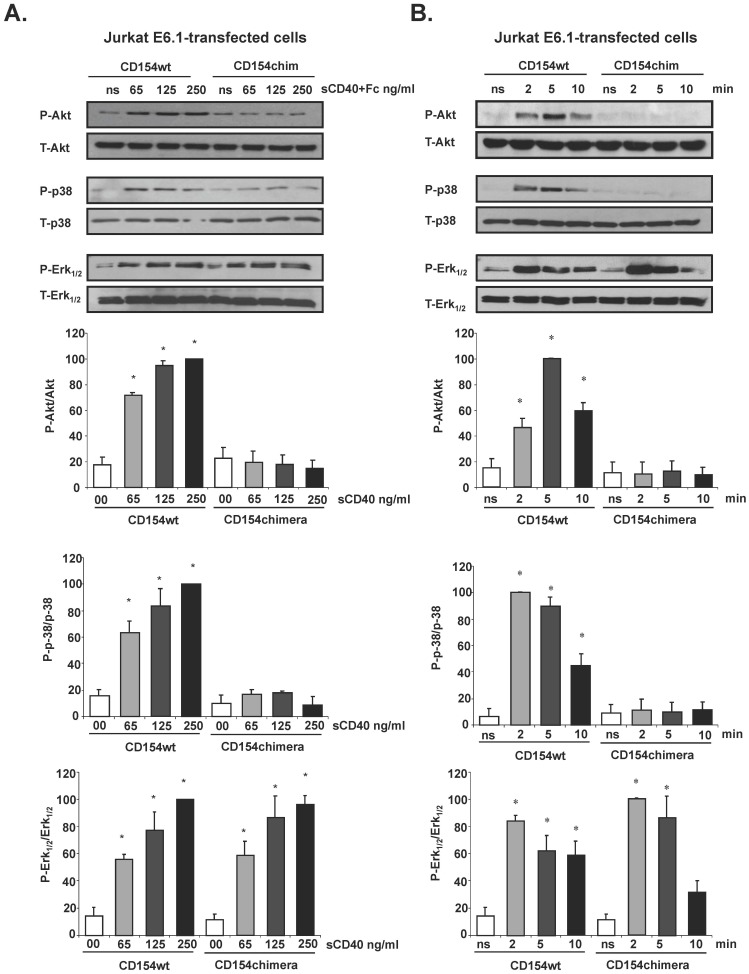
CD154-mediated Akt and p38 activation is dependent upon CD154 translocation into lipid rafts. *(A)*. Jurkat E6.1 cells stably transfected with CD154wt (CD154wt) or CD154chim (CD154chim) were stimulated for 5 minutes at 37°C with increasing concentrations of sCD40-Fc, or left unstimulated (NS), then lyzed and analysed by immunoblot to determine the activity of Akt, p38 and ERK1/2, using phospho-specific antibodies. *(B)*. E6.1-CD154wt and E6.1-CD154chim were stimulated with sCD40-Fc (250 ng/ml) or left unstimulated (NS) for the indicated times, then cells were lysed and analysed as in A. Densitometry was performed on western blots. Bars represent the percentage of protein phosphorylation (n = 4; *P<0.05 vs 00 for A) and ns for B)).

We have previously demonstrated that the biochemical disruption of lipid raft integrity abrogates the CD154-mediated activation of PKC and p38 MAPK. Here, we wanted to directly assess the role of lipid rafts in other CD154-induced signals using the chimeric form of CD154. Jurkat E6.1 cells stably expressing CD154wt or CD154chim were stimulated for 5 min with increasing concentrations of sCD40-Fc, and assessed for phosphorylation of Akt, and p38 and ERK1/2 MAPKs. Ligation of CD154wt, even at low CD40 concentrations (65 ng/ml), induced activation of ERK1/2, p38, as well as Akt, while engaged CD154chim was only capable of stimulating ERK1/2 phosphorylation (Fig. 3A). In a second set of experiments, Akt, p38, and ERK1/2 phosphorylation was investigated in transfected Jurkat E6.1 cells stimulated with 250 ng/ml of sCD40-Fc for different time points. Engaged CD154wt was capable of inducing Akt and p38 activation as early as 2 min, and this response was maintained for up to 5 min following stimulation with sCD40-Fc (Fig. 3B). For cells expressing CD154chim, stimulation with sCD40-Fc failed to induce Akt or p38 activation at all time points tested. On the other hand, ERK1/2 phosphorylation peaked 2 min following ligation with both CD154wt and CD154chim, following which an important decrease was exhibited (Fig. 3B). Altogether, data from these mutagenesis experiments strongly support our pervious results suggesting a role for lipid rafts in CD154-mediated signaling, and add Akt phosphorylation to the list of intracellular pathways triggered by the association of CD154 to lipid rafts.

### The CD154 Transmembrane Domain has no Effect on CD40 Binding

**Figure 4:**
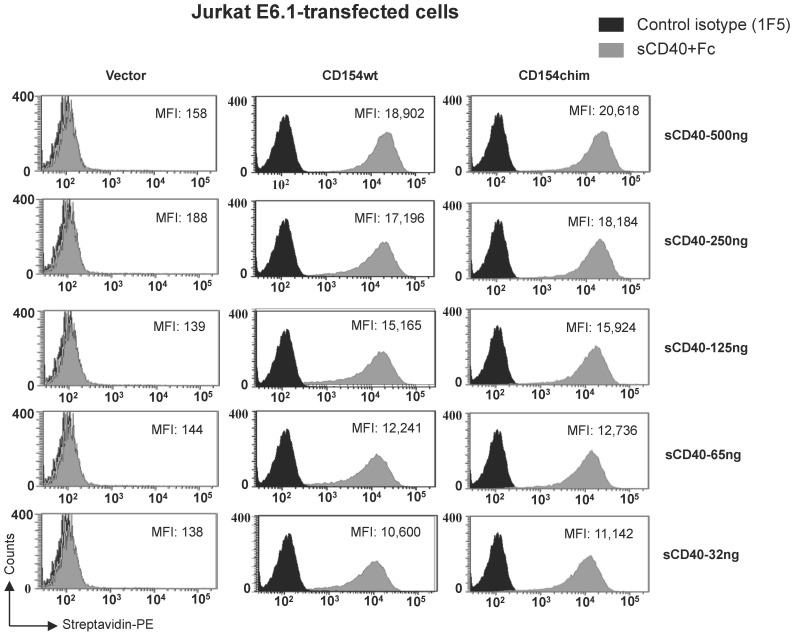
CD154 wild type and CD154 chimera bind similarly to soluble CD40. To assess their binding capacities, transfected cells were incubated with different concentration of biotin-conjugated soluble CD40-Fc (sCD40-Fc) (grey plot) for 30 min on ice and then, cells were analysed by flow cytometry. Mean fluorescence intensity (MFI) values are indicated.

To determine that the altered signaling events observed upon stimulation of CD154chim were not due to a decreased affinity to the receptor, we evaluated the binding capacity of biotin-conjugated sCD40-Fc to CD154wt and CD154chim-transfected cells. Fig. 4 shows that sCD40-Fc exhibited similar binding to both CD154wt and CD154chim-transfected cells (at concentrations ranging from 32 ng to 500 ng), suggesting that the CD154 transmembrane domain does not influence the binding of the molecule to its receptor. These results indicate that the abolished Akt and p38 responses observed upon ligation of CD154chim are due to failure of the molecule to translocate to lipid rafts rather than a decreased affinity to its receptor.

### The Association of CD154 to Lipid Rafts Enhances IL-2 Production in Co-stimulated T Cells

**Figure 5:**
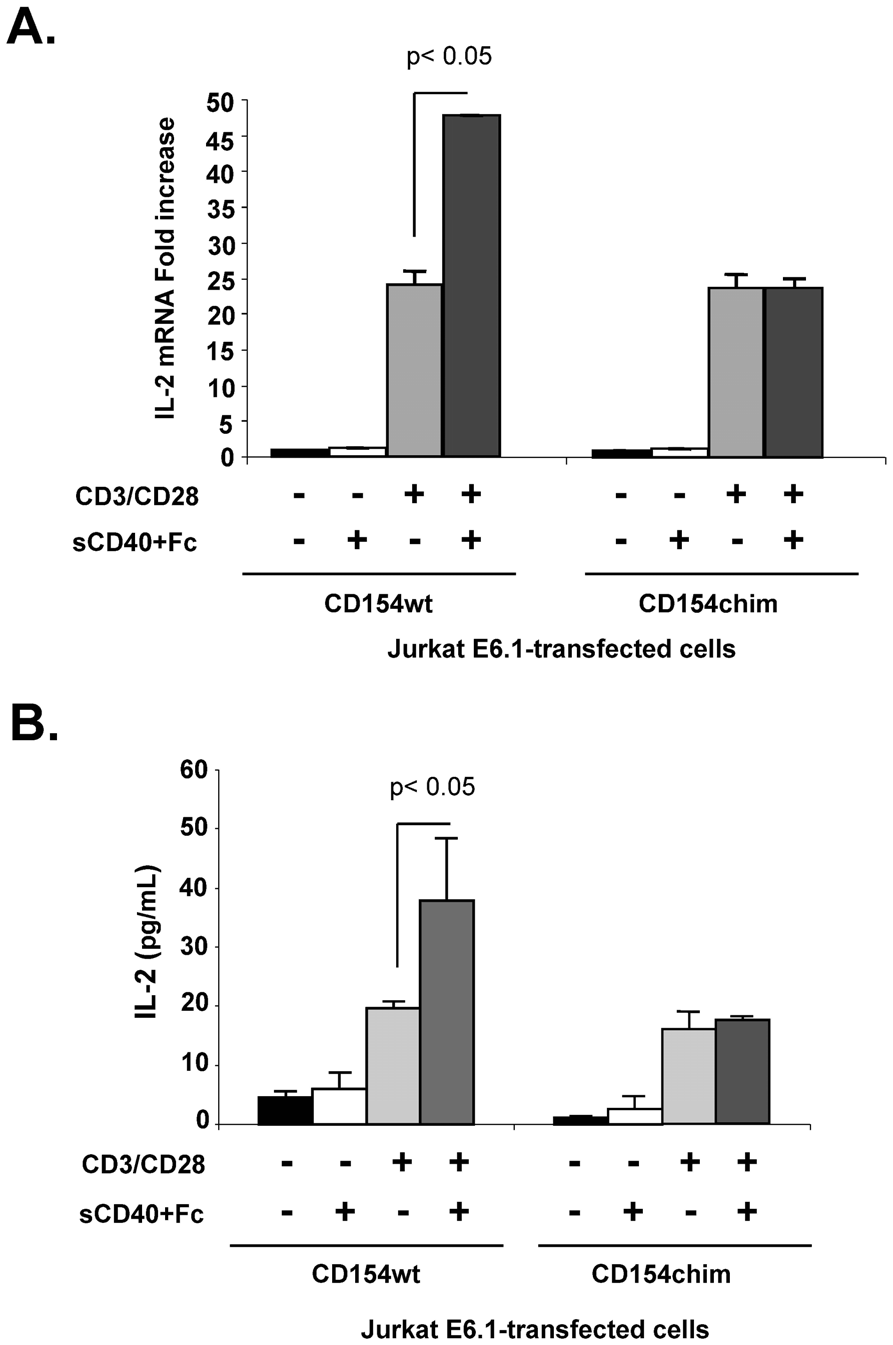
CD154/lipid raft association enhances IL-2 production in co-stimulated T cells. Jurkat E6.1-CD154wt and E6.1-CD154chim cells were stimulated with anti-CD3/CD28 in the presence or absence of sCD40-Fc for 5 h at 37°C. *(A)*. IL-2 gene expression in cells was assayed by RT-PCR. *(B)*. IL-2 protein levels in supernatants were determined by ELISA. Results are expressed as mean ± SEM from three independent experiments.

As the translocation of CD154 into lipid rafts appears indispensable for CD154-mediated Akt and p38 activation, we investigated next the biological relevance of this phenomenon. We have previously demonstrated that stimulating CD154 induced IL-2 production in T cells co-stimulated at their T cell receptor (TCR) [15]. Therefore, we aimed herein at investigating the role of lipid rafts in CD154-induced IL-2 production using T cells transfected with wild type or chimeric forms of CD154. Indeed, Jurkat E6.1 cells were either unstimulated, or stimulated with anti-CD3/antiCD28, with CD40, or with both stimuli. Activating cells expressing CD154wt or CD154chim via CD3/CD28 molecules induced IL-2 production at the mRNA and protein levels (Fig. 5A and 5B). Co-stimulation of T cells expressing CD154wt with anti-CD3/CD28 together with sCD40-Fc significantly enhanced IL-2 mRNA as well as protein levels. However, in cells transfected with CD154chim, co-activation with sCD40-Fc failed to potentiate CD3/CD28-mediated IL-2 production (Fig. 5). Our findings indicate that translocation of stimulated CD154 into lipid rafts is necessary for its role in promoting some immune functions, namely IL-2 production.

## Discussion

In our present study, we investigated the role of lipid rafts in CD154 signaling. Using a truncated form of CD154 lacking the cytoplasmic domain, we demonstrated that the translocation of CD154 into lipid rafts upon stimulation with its CD40 receptor is independent of CD154’ intracellular domain. On the other hand, using a CD154 chimeric mutant, in which the transmembrane domain was replaced by that of transferrin receptor I, a molecule known to be excluded from lipid rafts, we showed that the transmembrane domain of CD154 is required for its association with this compartment. In addition, our results show that the translocation of stimulated CD154 into lipid rafts is essential for some CD154-mediated intracellular signaling events, namely the activation of Akt and p38 MAPKs, but not ERK1/2 phosphorylation. CD154/lipid raft associations are also crucial for enhancing IL-2 production in T cells costimulated with CD3/CD28 and CD154 molecules.

We and others have previously reported the translocation of CD154 into lipid rafts upon ligation with its receptor CD40 [15], [20]. In the present study, using molecular techniques, whereby a mutation in the transmembrane domain of CD154 was conducted to inhibit CD154/lipid raft associations, we provide solid support for our previously reported finding where lipid raft disruption was undertaken using a biochemical treatment [15]. Mutagenesis techniques to abrogate the translocation into lipid rafts have a clear advantage over biochemical disruption of these domains using cholesterol-chelating agents [18]. Indeed, chemical agents that alter lipid rafts also disrupt other membrane molecules such as phosphatidylinositol-biphosphate [21], a substrate for a number of signaling proteins and a key element in many cellular events including membrane trafficking, cell polarization, ion channel regulation and cytoskeletal assembly [22]. Therefore, altering intracellular signaling upon treating cells with cholesterol-binding agents to disrupt their lipid raft integrity could also influence the disruption of important signaling molecules on the plasma membrane. Moreover, disrupting lipid rafts using the cholesterol-chelating agent methyl-β-cyclodextrin was shown to equally extract cholesterol from triton-insoluble membrane domains and from other cellular fractions [23]. Most importantly, the use of chemical agents to abrogate lipid rafts is limited to transient responses, as prolonged treatment with these agents (60–120 min) significantly reduces cell viability [24]. In addition, if extracted from the culture media, plasma membrane cholesterol can get reconstituted from intracellular stores [23] or from culture media, restoring as such lipid raft formation. Hence, biochemical agents that disrupt lipid rafts should be used with caution and mutagenesis approaches provide stronger and more reliable evidence for the role of lipid rafts in cell biology.

Other immunological molecules and receptors have been shown to associate with lipid rafts upon their oligomerization and ligation to their binding partners. Indeed, cross-linking TCR with anti-CD3 Abs revealed a colocalization of the receptor with aggregated lipid rafts [25]. Also, engagement of the multichain immune recognition receptor FcεRI was shown to upregulate its expression in the detergent non-soluble portion of the membrane [26]. Moreover, it was reported that cross-linking of BCR enhances its recruitment to lipid raft domains [27].

Most transmembrane proteins are excluded from lipid rafts, as they do not fit into these lipid-ordered structures [28]. However, few of such proteins exert their biological functions through localization into raft domains. To identify how raft targeting is occurring, mutagenesis techniques have been used, whereby intracellular, extracellular or transmembrane domains of proteins have been deleted or replaced [28]. For instance, targeting of CD44 and CD40 into lipid rafts is mediated by their transmembrane domain [18], [29], [30]. The results presented herein identify the transmembrane domain of yet another transmembrane protein, CD154 as being implicated in the association to lipid rafts.

Interestingly, our results show that the cytoplasmic domain of CD154 is not implicated in the translocation of the molecule into lipid rafts. Even though the intracellular portion of CD154 is void of intrinsic signaling motifs [15], associated signaling pathways may still be dependent upon this domain, as is the case for the CD40 receptor. Indeed, this hypothesis was recently confirmed by Kiani-Alikhan *et al.*, who demonstrated that a mutation in the intracellular domain of CD154 is associated with T-cell-dependent abnormalities in targeted patients [31]. Indeed, these patients present with an altered B cell phenotype, suggestive of a failure to form germinal centers where T cell-dependent B cell responses usually develop. In addition, affected subjects were capable of mounting an antibody response against T cell-independent meningococcal polysaccharide antigens, while they failed to produce specific antibodies against the tetanus vaccine, a T-cell-dependent protein antigen. The authors argued that such T-cell- dependent abnormalities are due to the absence of a signal through CD154 to T cells and the absence of germinal center formation [31]. Given these findings, our data showing that the cytoplasmic domain of CD154 is not implicated in the translocation of the molecule into lipid rafts upon stimulation strongly suggest that CD154/lipid raft associations occurs independently of signaling events. Such a phenomenon was also reported for CD40, where lipid raft translocation of the receptor did not require intracellular signaling [18].

An interesting finding outlined in our present study is the crucial role of lipid rafts in signaling induced via CD154. For many membrane-bound molecules, translocation into lipid rafts was shown to be necessary for intracellular signal transduction, as well as subsequent biological functions [26], [28]. Indeed, many signaling effectors such as Src family kinases including the lymphocyte-specific protein tyrosine kinase (Lck) and Lyn, which are involved in the activation of the multichain immune recognition receptors TCR, BCR and FcγRI, have been shown to localize in membrane rafts. Upon engagement of these receptors, their translocation into lipid rafts allows receptor activation and downstream intracellular signaling. For example, upon engagement, TCR translocates into lipid rafts where it can be activated by Lck protein tyrosine kinases. These kinases phosphorylate the immunoreceptor tyrosine-based activation motifs of TCR, thereby allowing the recruitment of the zeta-chain-associated protein kinase 70, which in turn phosphorylates important downstream substrates [32], [33], [34]. In the CD154 system, our group has previously demonstrated a role for lipid rafts in signaling mediated via CD40. Using mutagenesis techniques and a chimeric form of CD40, which lacks raft translocation capabilities, we have shown that CD40-mediated Akt and PI3K activation and the subsequent upregulation of CD80 in B cells is dependent on the association of CD40 with lipid rafts [18]. In this study, we clearly demonstrate, also using mutagenesis technique, that the translocation of CD154 to lipid rafts is required for downstream signal transduction, namely Akt and p38 MAPK activation. Interestingly, ERK1/2 phosphorylation was independent of CD154/lipid raft association. It is highly likely that the interaction of CD40 with CD154 localized in non-raft domains is activating the ERK1/2 pathway. Taken together, these findings outline an important role for lipid rafts in recruiting engaged membrane-bound CD154 molecules and enhancing some signaling events to ensure adequate subsequent biological functions.

In this matter, we show that the translocation of CD154 to lipid rafts upon engagement is required to enhance IL-2 production in T cells co-activated with CD3/CD28 molecules. These results are in synergy with the CD154/lipid raft-mediated signaling events described above, namely Akt and p38 activation. Indeed, costimulating T cells through TCR and CD28 activation induces IL-2 production via a p38 MAPK-dependent pathway [35], [36]. On the other hand, CD28-mediated Akt activation in T cells was shown to upregulate IL-2 production, by stimulating phosphorylation and nuclear export of NF90, an AU-rich element-binding protein that mediates IL-2 mRNA stabilization [37]. Thus, CD154-mediated IL-2 upregulation, which was found to be dependent on CD154/lipid raft association, likely represents a biological consequence of Akt and p38 activation in response to CD154 ligation. A CD154-mediated IL-2 upregulation was previously demonstrated by our group in TCR-activated T cells [15], [17]. However, the role of lipid rafts in this biological function had not been reported.

In conclusion, our findings confirm the key role of lipid rafts in CD154 signaling, and outline specific CD154-mediated intracellular events dependent on the translocation of CD154 into these raft domains. Given the recent description of new receptors for CD154, namely the αIIbβ3, α5β1, and αMβ2 integrins [4], [5], [6], the biological significance of CD154/lipid raft localization and its role in CD154 signaling should also be investigated upon CD154 binding to these receptors. Taken together, these data will identify new mechanisms involved in CD154 signaling that could represent interesting targets for therapeutic approaches against CD154-mediated diseases.

## Materials and Methods

### Antibodies and Reagents

The hybridoma producing antibodies raised against human CD154 (mAb C4.14) were produced in our laboratory. Murine IgG2a subtype (1F5) was a kind gift from Dr. Deans JP (Calgary, Canada). Polyclonal antibodies raised against Akt, p38, ERK1/2 and their anti-phosphorylated counterparts were purchased from Cell Signaling (Beverly, MA, USA). Secondary antibodies coupled to horse radish peroxidase (HRP) were from Santa Cruz Biotechnology. The cholera toxin B subunit conjugated to HRP (CTB-HRP) was obtained form Sigma-Aldrich. Human soluble CD40 (sCD40-Fc) was generated in our laboratory as described previously [15]. Biotin-labelled C4.14, 1F5 and CD40-Fc were prepared using the method provided by Pierce (Rockford, IL, USA).

### Cell Lines

Jurkat E6.1 T lymphocytes (obtained from ATCC, Rockville, MD, USA) stably expressing human wild-type CD154 (CD154wt), CD154 truncated mutant (CD154Δcyto), CD154 chimera (CD154chim), or empty vector (vector) were cultured at 37°C under a humidified 5% CO_2_ atmosphere in RPMI 1640 containing 5% fetal bovine serum (FBS), L-glutamine, 100 units/mL penicillin, 100 µg/mL streptomycin (Wisent, Montreal, QC, Canada) and supplemented with 100 µg/mL Zeocin (InvivoGen, Cederlane Laboratories, Burlington, Canada). A20 murine B cells (from ATCC) stably expressing human CD40 wild-type or empty vector were maintained in RPMI 5% FBS supplemented with 400 µg/mL hygromycin B (Wisent, Montreal, QC, Canada).

### Mutagenesis and Cell Transfection

Human CD154 wild-type was extracted from pcDNA3.1-hCD154 (a kind gift from Dr. Daniel Yung, Hema-Quebec, QC, Canada) and cloned into the KpnI-NotI restriction sites of pCDNA4-TO-myc-HisA Zeocin (Invitrogen). Human chimeric CD154 was generated by replacing the transmembrane domain of human CD154 by the transmembrane domain of transferrin receptor 1 (both are type II membrane proteins) as described below. First, the transmembrane domain of transferrin receptor 1 (TM-TFR1) containing parts of CD154 was amplified using the following primers: 5′-TATGGGACTA TTGCTGTGATCGTCTTTTTCTTGATTGGATTTATGATTGGCTACTTGGGCTAT-3′and 5′-ATAGCCCAAGTAGCCAATCATAA ATCCAATCAAGAA AAAGAC GAT CACAGCAATAGTCCCATA-3′. The extracellular domain of CD154 containing part of TM-TFR1 was amplified using the following primers: 5′-CTTG GGCTATCATAGAAGGTTGGAC-3′ and 5′-GATTTATG ATTGGCT ACTT GGG CTATCATAGAAG-3′. The intracellular part of CD154 containing part of TM-TFR1 was amplified with the following primers: 5′CAATAGTCCCATATTTCATGCTGATGGGCAG-3′ and 5′-CGATCACAGCAATAGTCCCATATTTCATGC-3′. Next, the extracellular domain of CD154 was fused with the TM-TFR1 using the following primers: 5′-CTTG GGCTATCATAGAAGGTTGGAC-3′ and 5′-ATAGCCCAAGTAGCCAATCATAA ATCCAATCAAGAA AAAGAC GAT CACAGCAATAGTCCCATA-3′, and then the fragment containing the extracellular domain of CD154 and TM-TFR1 was fused with the intracellular domain of CD154 using the following primers: 5′-CTTG GGCTATCATAGAAGGTTGGAC-3′ and 5′- CGATCACAGCAATAGTCCCATATTTCATGC-3′. The resulting chimera was subcloned into the pCDNA-4 plasmid using the KpnI and NotI restricted sites and verified by sequencing. CD154 lacking its cytoplasmic domain (CD154Δcyto) was generated by amplifying the transmembrane and extracellular domain of CD154 using the following primers: 5′ –ATTTGCGGCCGCATGATTTTTATGTATTTACTTACTGTT-3′ and 5′-ATTTGCGGCCGCTCAGAGTTTGAGTAAGCCA-3′. The resulting CD154Δcyto was subcloned into the pCDNA-4 plasmid using the KpnI and NotI restricted sites and verified by sequencing. Jurkat E6.1 T cells (8×10^5^ cells/sample) were transfected by electroporation with 3 µg of plasmid cDNA (CD154wt, CD154Δcyto or CD154chim) using the Neon transfection system (Invitrogen). Cells expressing comparable levels of CD154 were sorted using a Facs Aria II_4_ cell sorter (BD Biosciences, Mississauga, Ontario, CA).

### Flow Cytometry Analysis

The expression of CD154 in Jurkat E6.1 T cells transfected with human CD154 constructs was examined with a biotin-conjugated C4.14 mAb for 30 min on ice, followed by Streptavidin-phycoerythrin (PE) (Invitrogen). To determine the ability of CD154 wild-type and chimera to bind sCD40-fc, Jurkat E6.1 transfected cells were incubated with biotin-conjugated sCD40-Fc at different concentrations for 30 min on ice, followed by Streptavidin-PE. After washing with PBS/2%FBS, immunofluorescence intensity was detected using LSRII flow cytometry.

### Isolation of the Lipid-raft Microdomains by Sucrose Gradient Centrifugation

For lipid-raft microdomains isolation, 5×10^6^ cells (Jurkat E6.1-CD154wt, Jurkat E6.1-CD154Δcyto or Jurkat E6.1-CD154chim) were incubated alone, or with sCD40-Fc (250 ng/10^6^ cells), for 10 min at 37°C. For stimulation with membrane-bound CD40, Jurkat E6.1 stably transfected with CD154wt, or CD154chim were co-cultured with A20 B cell line transfected with empty vector (Jurkat E6.1 cells would be unstimulated) or with A20 B cells stably expressing human CD40 (Jurkat E6.1 cells would be stimulated with membrane-bound CD40) at aration of 3/1, for 10 min at 37°C. After stimulation, cells were rinsed once with ice-cold PBS, and then resuspended in 400 µL of ice-cold 1% Triton X-100/TNE (pH7,5; 10 mM Tris, 150 mM NaCl, 5 mM EDTA) containing a protease inhibitor cocktail (Roche Applied science). Following 60 min incubation on ice, the lysates were combined with an equal volume of ice-cold 80% sucrose (w/v) in 1X TNE, and loaded into a centrifugation tube. The samples were then overlaid with 2,4 mL of 35% sucrose and 1 mL of 5% sucrose (w/v), both prepared in 1X TNE. The sucrose gradient samples were spun at 35,000 rpm in a Beckman L7–55 ultracentrifuge for 16 h at 4°C using SW60 Ti rotors. Eleven fractions (380 µL) were collected, beginning at the top of the gradient. Fraction 12 corresponded to an insoluble pellet containing the cytoskeleton. Western blotting was performed to assess the CD154 protein distribution for each fraction.

### Western Blotting

To measure the signal pathway through CD154, Jurkat E6.1 transfected cells (5×10^5^ cells) were incubated in serum-free medium for 2 h at 37°C, and then stimulated with different concentrations of sCD40-Fc for different time points. The stimulation was stopped by the addition of hot 2X SDS sample buffer containing 10% 2-Mercaptoethanol (Invitrogen), protease inhibitors, phosphatase inhibitors (Sigma) and orthovanadate. Samples were then heated for 5 min at 100°C, separated through a 10% SDS-PAGE gel under reducing conditions and proteins were transferred to Immibilon P membrane (Millipore). Membranes were then blocked in 5% milk/TBS (0,1%-Tween) for 60 min at room temperature and incubated with indicated primary antibodies (diluted at 1∶1000 for 16 h at 4°C). Next, membranes were washed 3 times with 1X TBS+0,1% Tween, and incubated with HRP-conjugated secondary antibody (against rabbit) for 60 min. Finally proteins were revealed using enhanced chemiluminescence (PerkinElmer, MA, USA).

### Dot Blot Analysis

To detect the GM1 rafts marker, 10 µL of each fractions were dotted on a Immibilon P membrane (Millipore), which was then blocked in 5% milk/TBS (0,1%-Tween) for 30 min at room temperature. The membrane was then incubated with HRP-CTB (1/2000) for 60 min at room temperature and developed with ECL reagents.

### Real-time RT-PCR

To detect IL-2 mRNA levels, Jurkat E6.1 transfected cells (5×105/well, in triplicate) were incubated with anti-CD28 (BD; Pharmingen) and anti-CD3 (clone UCHT-1; BD Pharmingen) at a final concentration of 0,5 µg/mL for 4 h in the presence or absence of sCD40-fc (250 ng/well). Total RNA from 5×10^5^ T cells was isolated using TRIzol (Invitrogen). The extracted RNA was then reverse transcribed into cDNA and quantified as previously described [5]. Levels of IL-2 mRNA were detected by real-time PCR using the Fast Start SYBER Green Kit (Roche) in the presence of 0,4 µM of IL-2 or S9 specific primers. Relative fold of IL-2 mRNA expression values were determined applying the ΔΔC_t_ method and normalized to S9. Sequences of primer used are as follows: IL-2; sense 5′- TACAACTGGAGCATTTACTG-3′; anti-sense 5′-GTTTCAGATCCCTTTAGTTC-3′, S9; sense 5′-CGTCTCGACCAAGAGCTGA-3′; anti-sense 5′-GGTCCTTCTCATCAAGCGTC-3′.

### Cytokine ELISA

For IL-2 production, Jurkat E6.1 transfected cells (2.5×105/well, in triplicate) were incubated with anti-CD28 (BD; Pharmingen) and anti-CD3 (clone UCHT-1; BD Pharmingen) at a final concentration of 0,5 µg/ml for 24 h in the presence or absence of sCD40-fc (250 ng/well). Levels of IL-2 in the supernatant were measured by enzyme-linked immunosorbent assay (ELISA), according to the manufacturer’s instructions (BD Biosciences, optEIA). All of the values are expressed as the mean of triplicates± standard error of the mean (SEM).

### Statistical Analysis

Data were analyzed using Prism Software (GraphPad Software) using a Student’s *t* test. p<0,05 was considered significant.
